# Editorial to the Special Issue “Advances in Optical Biosensors and Chemical Sensors”

**DOI:** 10.3390/bios14090447

**Published:** 2024-09-19

**Authors:** Flavio Esposito, Stefania Campopiano, Agostino Iadicicco

**Affiliations:** Department of Engineering, University of Naples “Parthenope”, Centro Direzionale Isola C4, 80143 Napoli, Italy; flavio.esposito@uniparthenope.it (F.E.); stefania.campopiano@uniparthenope.it (S.C.)

## 1. Introduction

Optical biosensors and optical chemical sensors are innovative analytical tools that utilize light-based techniques to detect and quantify a plethora of biological and chemical substances. These sensors are designed to provide rapid, sensitive, and often real-time analysis, making them unique in fields such as medical diagnostics, environmental monitoring, food safety, and industrial process control. Optical biosensors specifically focus on detecting biomolecular interactions by leveraging variations in optical features, such as fluorescence, absorbance, or surface plasmon resonance, to provide information about the presence and concentration of target biomolecules. Optical chemical sensors, on the other hand, are designed to detect chemical substances through similar optical methods, often using colorimetric or spectroscopic techniques to monitor changes in chemical composition or concentration. Both types of sensors are renowned for their high sensitivity, specificity, and the ability to perform non-destructive testing, which allows for the continuous monitoring of samples without the need for extensive preparation [1,2].

As the demand for more efficient, accurate, and non-invasive sensing technologies grows, optical biosensors and optical chemical sensors continue to play a crucial role in advancing analytical science and technology. In this context, the Special Issue “Advances in Optical Biosensors and Chemical Sensors” of Biosensors collects seven notable papers on the topic; one is a review article, and the remaining are original articles. In Section 2, the main points of each contribution are summarized along with a brief overview of the working mechanisms involved. Section 3 briefly summarizes the main advantages and weaknesses as well as highlighting the future trends.

## 2. Overview of Contributions

The contributions reported in this Special Issue can be organized into the following three main families, based on the optical phenomenon or technology employed for the development of the sensors: fluorescence, Raman spectroscopy, and fiber optics.

### 2.1. Fluorescence-Based Sensors

Fluorescence-based optical biosensors are devices that detect and measure biological analytes using the principle of fluorescence. These biosensors leverage the unique properties of fluorescent molecules to provide the sensitive, selective, and real-time detection of target molecules such as proteins, nucleic acids, cells, or small biomolecules. They are widely used in biomedical research, diagnostics, environmental monitoring, and food safety. Fluorescence occurs when a molecule (fluorophore) absorbs light at a specific wavelength (excitation), causing it to move to a higher energy state. When the molecule returns to its ground state, it emits light at a longer wavelength (emission). This emitted light is what is detected and measured in this type of sensor. Fluorophores are key components of these devices and can be organic dyes (e.g., fluorescein or rhodamine), fluorescent proteins (e.g., GFP or RFP), or quantum dots. The choice of fluorophore depends on factors such as photostability, brightness, and compatibility with the biological system being studied. The emitted fluorescence is detected using optical components such as filters, mirrors, and photodetectors (e.g., photomultiplier tubes or CCD cameras). The intensity of the emitted light correlates with the concentration of the target analyte, providing quantitative data [3,4].

Over the past sixty years, the creation of glucose biosensors for monitoring diabetes has been a major focus and is often the first application that comes to mind when discussing biosensors. In Contribution 1, L. Colvin et al. designed a glucose biosensor using Concanavalin A (ConA) paired with a fluorescent probe, which exhibited changes in fluorescence intensity based on solvatochromism, i.e., a reversible shift in the emission spectrum depending on the solvent polarity. ConA is noted for its ability to reversibly bind with glucose and mannose molecules through competitive binding. To adapt the device for subcutaneous implantation, small mannose molecules labeled with Cyanine 5.5 (Cy5.5) were utilized. This modification allowed for excitation in the far-red wavelength range, improving the penetration depth of light through the skin and enhancing the return of the emitted signal. Cy5.5–mannotetraose detected glucose within the range of 25 to 400 mg/dL. Therefore, it demonstrated the capability of detecting glucose concentrations within physiological levels, even at the elevated concentrations required for an implantable format, as well as with a consistent signal across various skin tones.

In Contribution 2, W. Du et al. developed a fluorescent probe using a coumarin-based compound to obtain the differential detection of different biothiols in water-based solutions, namely cysteine (Cys), homocysteine (Hcy), and glutathione (GSH). Biothiols are involved in many disorders in living organisms. By reacting with biothiols, products with different conjugated structures are generated, resulting in different emission peaks at different excitation wavelengths. Cys, Hcy, and GSH can be distinguished by analyzing their fluorescence and UV-Vis spectra and can be validated through mass spectrometry, achieving a limit of detection (LOD) of 0.02 μM for Cys, 0.42 μM for Hcy, and 0.92 μM for GSH, all of which were lower than the concentrations of these biothiols typically found in cells. Finally, the performance of the probe was evaluated in cellular assays with both naturally occurring and externally introduced biothiols.

In Contribution 3, the author presented a dual nanosensor for the detection of two important parameters in medical and industrial applications, i.e., O_2_ and pH. This sensor is based on organically modified silica (ormosil) nanoparticles, which were used as a matrix for hosting the two probes of O_2_ (Pt-TPFPP) and pH (salicylamide). When excited at 365 nm, the sensor produced blue fluorescence at 457 nm and red fluorescence at 648 nm, corresponding to salicylamide and Pt-TPFPP, respectively. These distinct emission peaks enabled the simultaneous analysis of pH within a range of 6 to 10, as well as oxygen levels from 0 to 100%.

The contamination of water sources by heavy metals presents a serious threat to human health and the environment, leading to numerous related risks. In Contribution 4, R. Ali et al. reported a chemosensor, based on an Isoxazolidine derivative (IXZD) embedded in a PVC film, for the detection of Hg(II) mercury ions. The suggested detection mechanism involves metal–ligand coordination between IXZD and Hg(II) ions, forming a metal complex. The probe also functions as a turn-on optical sensor for pH detection, as its luminescence significantly increases in the deprotonated state compared to the protonated state. The detection of mercury ions by the IXZD probe was analyzed using UV-Vis absorption and fluorescence emission measurements. The sensor exhibits a high sensitivity, selectivity, and reversibility, with a low LOD of 0.025 μM at pH 7.4.

### 2.2. Raman Spectroscopy Sensing

Raman spectroscopy and surface-enhanced Raman spectroscopy (SERS) are powerful analytical techniques that are used in optical biosensing. When light interacts with a molecule, most photons are elastically scattered (Rayleigh scattering), whereas a small fraction of the light is inelastically scattered (Raman scattering), resulting in a shift in the energy (and therefore the wavelength) of the scattered photons. Such a shift is employed in Raman spectroscopy techniques to provide information about the vibrational modes of the molecules, which are unique to specific chemical bonds and molecular structures [5]. SERS is an advanced version of Raman spectroscopy that greatly enhances the Raman scattering signal by several orders of magnitude (up to 10^6^ to 10^14^ times). This enhancement is achieved by adsorbing the target molecules on rough metal surfaces or nanostructures, such as silver, gold, or copper nanoparticles. The increased effect is mainly associated with either electromagnetic or chemical enhancements [6,7].

In Contribution 5, by P. Papaspyridakou et al., the authors reported about a Raman spectroscopy-based method for determining the concentration of ethanol and toxic alcohols (such as methanol and isopropanol) in beverages and spirits, which is essential for ensuring good health and for detecting counterfeit products. A portable Raman device was employed that achieved several benefits, including non-destructive and non-invasive analysis for in-line monitoring on a production line. The calibration curves for the relevant alcohols were obtained and verified. The LODs were calculated and found to be below the legal thresholds. The impact of the liquor color, as well as the bottle color, shape, and thickness, were examined. Several alcoholic products were analyzed, and the results were compared with the nominal values listed on the bottle labels.

Contribution 6, by D. Kotturi et al., investigates personalized medicine, where the continuous or on-demand monitoring of metabolites to adjust medication dosages in real time is of vital importance. In this scenario, surface-enhanced spatially offset Raman spectroscopy (SESORS) is an optical method that can detect SERS-active substances beneath a barrier, allowing for the frequent monitoring of metabolites. They examine how the signal intensity changes spatially as it passes through tissue, using both experimental approaches and Monte Carlo modeling. A hydrogel containing SERS-active material, similar in size to an implant, was positioned beneath tissues of varying thicknesses. Raman spectra were collected at the air–tissue interface across different offsets from the excitation point. Raman signals were detected through all tissue thicknesses; both the computational model and experimental data show the highest signal intensities at a 0 mm offset, and a steep decline in intensities was observed as the offset increases. These findings differ from previously reported SORS studies, which involved larger targets than implants.

### 2.3. Fiber Optic-Based Sensors

Fiber optic-based optical biosensors are a type of biosensor that utilizes optical fibers to transmit light signals for the detection of biological analytes. These biosensors combine the sensitivity and specificity of optical detection methods with the unique properties of optical fibers, such as their flexibility, small size, and ability to transmit light over long distances with minimal loss. They are widely used in various fields, including medical diagnostics, environmental monitoring, food safety, and biochemical research as they can withstand harsh environmental conditions [8].

In this scenario, there are different sensing mechanisms that are worth mentioning. Surface plasmon resonance (SPR) biosensors utilize optical fibers coated with a thin layer of metal (typically gold or silver) or metal nanoparticles (in this case, it is referred to as localized surface plasmon resonance or LSPR) to detect changes in the refractive index near the fiber surface. When light propagates through the fiber, it excites surface plasmons at the metal–dielectric interface. The binding of the target analyte to the surface changes the local refractive index, resulting in a shift in the SPR signal, which can be measured to determine the concentration of the analyte [9,10]. Interferometric biosensors use the interference pattern created by combining two or more light beams traveling through different paths of the optical fiber. When an analyte binds to the biorecognition element on the fiber, it causes a change in the optical path length or phase shift of the light, which is detected as a change in the interference pattern. Common types include Mach–Zehnder interferometers (MZIs), Fabry–Pérot interferometers (FPIs), and Michelson interferometers (MIs) [11,12]. Evanescent wave biosensors rely on the interaction of the evanescent wave, which extends beyond the core of the optical fiber into the surrounding medium, with the target analyte. When an analyte binds to the sensor surface, it alters the evanescent wave properties, leading to measurable changes in light intensity or wavelength; examples include etched or tilted fiber Bragg gratings (FBGs) [13,14], long period gratings (LPGs) [15,16], and lossy mode resonances (LMRs) [17,18].

The review by the group of S. Kumar (Contribution 7) provides a comprehensive illustration of the conventional and novel tapered optical fiber sensor structures used in chemical and biological sensors. The authors considered different technological solutions based on SPR/LSPR, MZIs, and evanescent wave devices by also considering fabrication methods and signal demodulation techniques. Finally, they present several applications ranging from chemical sensing to gas sensing to biosensing.

## 3. Conclusions and Outlooks

This Special Issue looks at the recent advancements and emerging trends in optical fiber chemical sensors and biosensors, highlighting the latest progress in theory, design, fabrication, and application or validation. The high-quality articles offer a valuable insight into the current state of the field and its future directions. The authors primarily explored three distinct optical technologies and methods, focusing on developments mainly for medical applications, as well as for industrial and environmental monitoring.

In particular, it is worth highlighting that fluorescence-based optical biosensors are powerful tools offering high sensitivity, specificity, and versatility for detecting and analyzing a broad spectrum of biological and chemical analytes. While there are challenges to their use, ongoing advances in fluorophore design and data analysis are expanding their capabilities and applications. Raman spectroscopy and SERS are powerful techniques for the detection and characterization of biomolecules. While Raman spectroscopy provides a molecular fingerprint for qualitative and quantitative analysis, SERS enhances the sensitivity to the single-molecule level, opening new possibilities for early disease detection, environmental monitoring, and food safety. Despite some challenges, ongoing advances in substrate design, data analysis, and instrumentation miniaturization are expanding the applications and capabilities of these techniques in various fields. Finally, fiber optic-based biosensors show a high sensitivity, specificity, flexibility, and ability to provide real-time remote sensing, making them suitable for various applications from medical diagnostics to environmental monitoring. Despite the challenges related to fabrication, fragility, and surface fouling, ongoing advancements in materials, nanotechnology, and the portability of the setups continue to enhance their performance and broaden their application scope.

## Data Availability

Not applicable.
